# Descriptive survey of women’s childbirth experiences in two state hospitals in KwaZulu-Natal

**DOI:** 10.4102/curationis.v44i1.2164

**Published:** 2021-04-29

**Authors:** Uwonkunda P. Mutabazi, Petra Brysiewicz

**Affiliations:** 1 School of Nursing and Public Health, University of KwaZulu-Natal, Durban, South Africa

**Keywords:** childbirth, childbirth experience, Childbirth Experience Questionnaire, KwaZulu-Natal, maternal healthcare

## Abstract

**Background:**

Giving birth is one of the most important events in a woman’s life and is a highly individualistic and unique experience.

**Objectives:**

The study aimed to describe women’s childbirth experiences in two state hospitals in KwaZulu-Natal.

**Method:**

A non-experimental, quantitative, descriptive survey of low-risk mothers was conducted in two state hospitals by using the Childbirth Experience Questionnaire (CEQ).

**Results:**

With a response rate of 96%, 201 questionnaires were completed and returned. The highest mean score of the four dimensions of the CEQ was for the dimension of Professional Support (3.1). The results of the individual dimension items scoring the highest positive response were: *I felt that I handled the situation well* (147; 74%) (Own Capacity); *I felt very well cared for by my midwife* (165; 82%) (Professional Support); 151 respondents (76%) scored the item *My impression of the team’s medical skill made me feel secure* as the highest positive experience (Perceived Safety); and *I felt I could have a say in the choice of pain relief* (105; 52%) (Participation). The relationship between demographic variables (age, level of education, parity, antenatal clinic attendance, induction of labour, augmentation and duration of labour) and respondents’ scores of the CEQ dimensions was calculated, and only the dimension of Perceived Safety and duration of labour (≥ 12 hours) were found to be significant (*p* = 0.026).

**Conclusion:**

From the women perspectives, the study results described childbirth experience as multi-dimensional experience and subjective. Both positive and negative experiences coexisted in all dimensions of the CEQ, with the dimension of Professional Support scoring the highest positive response. To maintain a positive birth experience, the study suggests that women should be involved and equipped with knowledge on the process of childbirth.

## Introduction and background

Giving birth is one of the most important events in a woman’s life, and it is a highly individualistic and unique experience (Namujju et al. 2018). Childbirth is associated with physical, physiological and psycho-social changes in women’s life, requiring support and care, not only from professional carers but also from their family members (Lunda, Minnie & Benade 2018). Childbirth support offers women the opportunity of having a positive childbirth experience and outcomes (Maputle 2018). This is very important, especially for first-time mothers, as this support, help and motivation assist them in developing good self-esteem and positive feelings towards the baby and enable an easier adjustment to the role of motherhood and future pregnancies (Nilsson et al. 2013).

The experience of giving birth, whether positive or negative, is remembered for a lifetime and can have long-term implications on a woman’s health and well-being (Karlström, Nystedt & Hildingsson 2015; Mohammad et al. 2014; Sengane 2013).

Women’s experiences of childbirth are influenced by a number of factors, for example attendance of antenatal classes, level of labour pain, medical intervention, support received from partners and professionals and a sense of being in control (Nilsson et al. 2013). The positive factors, such as a sense of being in control of what is happening and being involved in decision-making, are said to increase women’s self-confidence and feelings of accomplishment, thus ensuring a better adjustment to motherhood and the development of a strong bond with her baby (Namujju et al. 2018). However, negative experiences, such as a medicalised birth environment and unplanned obstetrical emergency interventions, have been found to contribute to emotional upsets and fear of future pregnancies and childbirth. This may lead to post-partum depression and reduce the care given to the baby by the mother (Nilsson et al. 2013; Størksen et al. 2013).

To date, literature has primarily addressed isolated aspects of childbirth and focussed on quality of care, risks and complications of childbirth (Dencker et al. 2010; Nilsson et al. 2013). Experience encountered by women during childbirth has received little attention, especially in low- and middle-income countries where greater priority has been assigned to preventing pregnancy-related deaths (Nilsson et al. 2013; Fisher et al. 2012). Across the South African health care system, enquiry about women’s experience of childbirth is limited (Hastings-Tolsmaa, Nolteb & Temane 2018), and knowledge of women’s birth experiences is thus important (Dencker et al. 2010).

## Aim

This study aimed to describe women’s childbirth experiences in two state hospitals in KwaZulu-Natal (KZN).

## Methods

A non-experimental, quantitative descriptive survey was used as this provides a broad view of the population through a study of a representative sample.

### Research setting

The study was conducted in two selected state hospitals located in the eThekwini District, KZN. These hospitals are both regional and referral hospitals in KZN, serving a large population from different surrounding communities and having large and busy maternity departments.

Hospital A is a large teaching hospital, providing a tertiary service for the entire province of KZN and part of Mpumalanga and Eastern Cape. It has departments comprising both outpatient and inpatient units within different specialities, including obstetrics and gynaecology.

Hospital B is a state district and regional hospital situated in the peri-urban area of the eThekwini District of KZN. It is a hospital where obstetrics and gynaecology services are offered at a district level. It is also a referral hospital for 16 clinics in its catchment area, and this contributes to the high number of childbirths for this hospital.

### Population, sample and sampling

Purposive sampling was used whereby the respondents were selected according to the criteria that were relevant to the topic. Purposive sampling enabled the researcher to obtain useful information as the respondents had the characteristics of the population being studied. The study population included all post-partum patients in the two hospitals, meeting the following inclusion criteria: (1) women who were 18 years or older, (2) who had a normal vaginal delivery of a live and healthy infant and (3) who were already discharged from hospital and waiting to go home. A statistician was consulted to assist with the determination of sample size, and the sample of a minimum of 185 respondents was determined as adequate based on monthly statistics of deliveries in both hospitals. The sample size was calculated by using the effect size and the desired statistical power.

### Data collection tool

The Childbirth Experience Questionnaire (CEQ), developed by Dencker et al. (2010), was chosen for this study as it measures different important dimensions of the childbirth experience, with a higher dimension score indicating a better childbirth experience. Permission to use and translate the questionnaire was obtained from the developers. The questionnaire was translated into isiZulu by professional language translators at the university.

This structured questionnaire has 22 items in total and is made up of 19 items representing four dimensions of a childbirth experience, namely *Own Capacity* (six items), *Professional Support* (five items), *Perceived Safety* (five items) and *Participation* (three items). These items were originally scored by using a 4-point Likert scale, which has the following options: strongly agree, agree, disagree and strongly disagree. To suit the context of the study, the CEQ was modified to include relevant demographic data of the respondents and the response format was transformed into a 5-point Likert scale on the advice of a statistician to include a neutral option, as increasing the response alternatives improves reliability and validity (Lozano, García-Cueto & Muñiz 2008). The 5-point Likert scale was as follows: strongly agree (5), agree (4), neutral (3), disagree (2) and strongly disagree (1). The higher dimension score indicates a better childbirth experience.

The remaining three items used a visual analogue scale (VAS) where the respondents were asked to mark on the line between two end points, their memory of labour pain, sense of security and control. The questionnaire also had an open-ended question at the end for any additional comments.

### Data collection process

Data were collected over a period of 2 months, after the ethical approval from the university and Department of Health and permission from the hospitals. Authorised by the postnatal unit managers and assisted by the nurses in the postnatal ward, the researcher distributed a detailed information letter and consent form to the women who met the criteria of the study and were discharged, waiting to be fetched by relatives. Those who were willing to participate in the study signed the consent form and completed questionnaires which were collected by asking the respondents to drop them into a sealed box.

### Data analysis

Descriptive statistical results were presented in the form of frequency tables and percentages by using SPSS-24. Inferential statistics by using the Mann–Whitney *U* test was used to determine the relationship between the demographic data (age, level of education, parity, antenatal clinic [ANC] attendance, induction of labour [IOL], augmentation and duration of labour) and the items of the CEQ. Significance was set as *p* < 0.05. The VAS scores were transformed to categorical values where 1 = No and 2 = yes and depending on where the respondent made a mark on the line. The open-ended questions were categorised (guided by the CEQ dimensions) and represented quantitatively. To establish the average dimension scores for the CEQ, the 5-point Likert scale was converted into an average out of 4. To then identify individual item scores, the 5-point Likert scale was condensed to form a 3-point Likert scale where responses of ‘strongly disagree’ and ‘disagree’ as well as ‘agree’ and ‘strongly agree’ were grouped together. Negatively worded items were reversed.

### Validity and reliability

The CEQ was found to be a valid and reliable measure of the childbirth experience in the UK population (Walker et al. 2015). Content validity of the questionnaire was assessed in the current study by midwifery and research experts at the university. The questionnaire was pilot-tested on five women meeting the inclusion criteria, three in Hospital A and two in Hospital B. Both English and isiZulu versions of the questionnaire were used for the pilot study, depending on the respondents’ preferences. No changes were made to the questionnaire after the pilot study, and these data were not included for analysis.

### Ethical considerations

The research was reviewed and approved by the Research Ethics Committee of the University of KwaZulu-Natal (reference number: HSS/0917/016M) and the KwaZulu-Natal Health Research Committee (reference number: HRKM228/16). Permission to conduct the study was obtained from the management of Hospital A and Hospital B. Prior to the commencement of data collection, respondents were given an information sheet explaining the study and then asked to provide written informed consent (available in either isiZulu or English). They were informed that their responses were confidential, data could not be traced back to them and their responses would not influence their treatment in any way.

## Results

The researcher (P.M.U.) handed out 210 questionnaires, and 201 questionnaires were completed and returned, giving a response rate of 96%. Some of the returned questionnaires had missing data; therefore, results were presented based on the number of responses available on each questionnaire item.

### Demographic data

The majority of the women were aged between 18 and 25 years (mean age was 26.8) and the majority had secondary education (Table 1). The gravidity of the respondents ranged from 1 (65; 32%) to 7 (1; 1%), with the majority being Gravida 2 (70; 35%). In relation to parity (para), the majority (77; 38%) were Para 1.

**TABLE 1:** Demographic data of respondents.

Characteristics	*n*	%
**Age (*n* = 200)**		
18 – < 25 years	84	42
25 – < 30 years	48	24
30 – < 35 years	46	23
35+ years	22	11
**Level of education (*n* = 195)**		
Primary	14	7
Secondary	118	61
Tertiary	63	32
**Gravidity (*n* = 200)**		
1	65	32
2	70	35
3	37	18
4	20	10
5	7	4
7	1	1
**Parity (*n* = 201)**		
1	77	38
2	70	35
3	33	16
4	16	8
5	3	2
6	2	1
**Attended antenatal classes (*n* = 199)**		
Yes	177	89
No	22	11
**Induction of labour (*n* = 197)**
Yes	62	31
No	135	69
**Augmentation of labour (*n* = 191)**
Yes	90	47
No	101	53
**Duration of labour (*n* = 198)**
< 12 h	135	68
≥ 12 h	63	32

### Childbirth Experience Questionnaire

**FIGURE 1:**
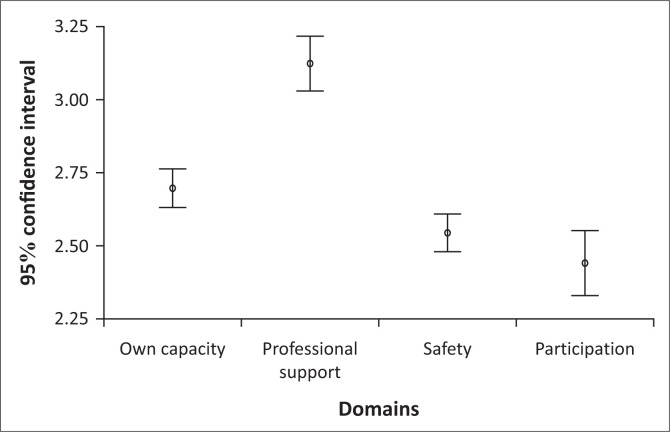
Mean dimension scores.

The results of the CEQ dimension scores are shown in Figure 1. The mean scores of the four dimensions were: 3.1 for *Professional Support*, 2.7 for *Own Capacity*, 2.5 for *Perceived Safety* and 2.4 for *Participation*. The highest mean score reflected was for the dimension of *Professional Support* (see Figure 1).

The results of the CEQ individual dimension items are shown in Table 2.

**TABLE 2:** Childbirth Experience Questionnaire.

Questionnaire item	Disagree		Neutral		Agree	
	*n*	%	*n*	%	*n*	%
**Own capacity**						
Labour and birth went as I had expected (*n* = 201)	54	27	31	15	116	58
I felt strong during labour and birth (*n* = 201)	53	26	43	22	105	52
I felt capable during labour and birth (*n* = 192)	39	20	34	18	119	62
*I was not tired during labour and birth* (*n* = 196)	85	43	25	13	86	44
I felt happy during labour and birth (*n* = 197)	73	37	32	16	92	47
**I felt that I handled the situation well (*n* = 200)**	25	12	28	14	**147**	**74**
**Professional support**						
My midwife devoted enough time to me (*n* = 201)	28	14	10	5	163	81
My midwife devoted enough time to my partner (*n* = 174)	43	25	42	24	89	51
My midwife kept me informed about what was happening during labour and birth (*n* = 200)	29	14	13	7	158	79
My midwife understood my needs (*n* = 199)	26	13	19	10	154	77
**I felt very well cared for by my midwife (*n* = 201)**	15	8	21	10	**165**	**82**
**Perceived safety**						
*I did not feel scared during labour and birth* (*n* = 200)	111	55	42	21	47	24
I have many positive memories from childbirth (*n* = 201)	35	17	38	19	128	64
*I do not have many negative memories from childbirth* (*n* = 197)	47	24	37	19	113	57
*Some of my memories from childbirth do not make me feel depressed* (*n* = 200)	54	27	24	12	122	61
**My impression of the team’s medical skill made me feel secure (*n* = 210)**	25	12	25	12	151	76
**Participation**						
I felt I could have a say in whether I could be up and about or lie down (*n* = 201)	77	38	42	21	82	41
I felt I could have a say in deciding my birthing position (*n* = 201)	87	43	39	20	75	37
**I felt I could have a say in the choice of pain relief (*n* = 201)**	64	32	32	16	**105**	**52**

Note: Italics highlights negatively worded statements which were reversed; Bold reflects highest mean score.

When considering the item in each dimension scoring the highest positive response (agree) *I felt that I handled the situation well* (147; 74%) was the highest in Own Capacity dimension. The Professional Support dimension showed the item *I felt very well cared for by my midwife* (165; 82%) as the most positive score. The dimension of Perceived Safety showed that 151 respondents (76%) scored the item *My impression of the team’s medical skill made me feel secure* as the highest positive experience. In the fourth dimension Participation, the item that had the highest positive score was *I felt I could have a say in the choice of pain relief* (105; 52%).

The results from the VAS showed that the vast majority of the respondents (92%) experienced pain during childbirth, 84% of the respondents indicated that they had experienced a sense of security and 64% of the respondents had control during childbirth.

The relationship between demographic variables (age, level of education, parity, ANC, IOL, augmentation and duration of labour) and respondents’ scores of the CEQ dimensions was calculated, and only the dimension of Perceived Safety and duration of labour (≥ 12 h) was found to be significant (*p* = 0.026).

### Open-ended questions

The results from open-ended questions revealed that positive childbirth experiences were associated with feeling supported and cared for by nurses and doctors, feeling safe and positive labour process and outcomes. Negative childbirth experiences were linked to the severity of labour pains, poor treatment by the staff, lack of privacy, poor sanitation, lack of control and communication. Responses to the open-ended question are illustrated in Table 3.

**TABLE 3:** Open-ended question.

Childbirth experiences	Category	Number of responses	Example of a response
Positive experiences	Feeling supported and cared for by nurses and doctors	5	‘I was well cared for, they treated me very good. I can’t complain about anything.’ (Hospital B, 25 years, G2, P2)
	Feeling safe	2	‘Nurses who were there delivered me well. They did not shout at me. I felt safe.’ (Hospital A, 30 years, G4, P4)
	Positive labour process and outcomes	4	‘…Unique and eye-opening experience. You feel vulnerable and need as much help as you can get. In the end it is rewarding and relieving to see your child alive.’ (Hospital A, 22 years, G1, P1)
Negative experiences	Severity of labour pain	2	‘During my labour it was very difficult. I never felt like that before … they must provide pain killers.’ (Hospital B, 30 years, G1, P1)
	Poor treatment by the staff	2	‘I suggest that doctors and nurses should stop treating people who are in labour as if they are not human, like shouting at them while they are feeling so much pain*….*’ (Hospital A, 19 years, G1, P1)
	Shortage of staff	2	‘I think if they increase the number of nurses, they will be able to give enough time to the patients.’ (Hospital B, 30 years, G1, P1)
	Lack of privacy	1	‘No privacy respected. I could see other women in the next bed and opposite me, the very same way they could see me. The curtains are not good to keep my privacy.’ (Hospital A, 35 years, G3, P3)
	Poor sanitation	2	‘The condition of the bathroom is scary; I may catch disease.’ (Hospital B, 30 years, G2, P2)
	Lack of control and communication	2	‘I was not aware of anything during and after birth. There is a need for better patient–staff communication skills to be maintained.’ (Hospital B, 24 years, G1, P1)

G, gravida; P, parity.

### Ethical considerations

The ethical clearance was received from the University of KwaZulu-Natal, Humanities & Social Sciences Research Ethics Committee (HSS/0917/016M).

## Discussion

The following discussion is based on the dimensions of childbirth experiences, and significant findings on different dimensions are highlighted, compared and discussed according to the literature related to the experience of childbirth.

### Professional support

The CEQ and the responses from the open-ended question supported the importance of the *Professional Support* dimension for a positive birth experience. This was supported by Zhu et al. (2019) who showed that the *Professional Support* dimension was rated the highest CEQ dimension in China, Spain, Sweden and England. Kordi, Bakhshi and Tara (2014) in Iran supported this and indicated that midwifery support provided the coping strategies needed to deal with childbirth stress, thereby enabling mothers to experience a more comfortable labour with less anxiety. According to Maputle (2018), a study conducted in South Africa found that providing support during childbirth could reduce the risks of medical interventions and increase a chance of women having positive initial breastfeeding experience. This allowed women to feel more empowered and increased their feelings of control and strength of their body, thus allowing for a better ability to manage the pain (Nilsson et al. 2013). However, when mothers’ expectations regarding midwives’ care during labour were not met, they become dissatisfied and may have negative experiences of their labour and feel abandoned (Nilsson et al. 2013; Sengane 2013). A systematic qualitative review supported the fact that what mattered the most to women was that the childbirth experience fulfilled or exceeded their prior personal and socio-cultural beliefs and expectations (Downe et al. 2018).

### Own capacity

The overall findings with regard to women’s experience of *Own Capacity* suggested that the majority of the women in the current study were confident in their ability to cope with the childbirth process. A study by Fair and Morrison (2012) in the USA showed that the mothers’ experiences of control significantly predicted birth satisfaction. Cook and Loomis (2012) in their study in Ontario, Canada, to identify the choice and control in women’s childbirth experiences also found that control over physical, emotional and mental aspects of childbirth was important for women’s satisfaction. Being in control was perceived as a positive component of labour, with a preponderance of women affirming that it was essential to maintain personal dignity during labour in order for them to achieve a positive childbirth experience (Ahmar & Tarraf 2014).

It is, however, important to note that during a prolonged labour, women have been found to lose their capacity to cope and abandon themselves to the physicians and midwives (Nystedt & Hildingsson 2014).

### Perceived safety

The respondents in the current study indicated the item *I have many positive memories from childbirth* as receiving the highest positive score in this dimension; however, previous studies have established childbirth to be scary and traumatising. Toohill et al. (2014) conducted a study in Australia and reported that women reported high levels of fear, with some experiencing moderate to severe levels of anxiety or depression. The competence and skill of the medical team responsible for the woman in labour are essential, and this allows woman to feel safe, which in turn contributes to a positive childbirth experience (Floyd et al. 2014; Kordi et al. 2014; Paudel et al. 2015; Renfrew et al. 2014; Mensah, Mogale & Richter 2014). The current study found that labour ≥ 12 h influenced the *Perceived Safety* of the women, and this was supported by Adams, Eberhard-Gran and Eskild (2012) who found that labour duration was significantly longer in women with a fear of childbirth when compared with women without the fear of childbirth. The respondents in the current study also described a lack of privacy during childbirth as well as hygiene concerns in the hospital as the contributing factors to feeling unsafe. Such issues regarding the lack of cleanliness and poor hygiene were also reported in a study conducted in a semi-rural district in South Africa by Zitha and Mokgotle (2020), who found that the respondents felt unsafe and feared that they were being exposed to diseases. The importance of privacy and the cleanliness of the childbirth environment was mentioned in a number of previous studies as a contributing factor to a positive childbirth experience. Paudel et al. (2015), in a study in Nepal, agreed with the need for privacy and cleanliness. The same findings were identified by Floyd et al. (2014) who examined women’s views and experiences of maternity care in Ghana (Mensah et al. 2014). Iravani et al.’s (2015) study findings also indicated the importance of the physical environmental conditions (a pleasant environment and clean rooms) and the assurance of privacy.

### Participation

This was the lowest-scoring dimension, and the majority of the women in this study indicated that they did not have a say in moving around or choosing a birthing position. A study carried out in Accra, Ghana, by Mensah et al. (2014) highlighted the importance of this aspect of childbirth with a theme emerging from their study of women’s experience of childbirth emphasising the importance of being in control during labour. Only 52% of the women in the current study felt that they could have a say in pain relief. A study in Tshwane District, South Africa, supported this result and also reported how women had been denied pain medication during labour (Malatji & Madiba 2020). A study conducted in Australia also showed that women reported fear regarding the childbirth process, and this was mainly because of the fear of the intensity of labour pain (Toohill et al. 2014).

## Recommendations

It is recommended that maternity healthcare workers should be aware of the problematic areas of the childbirth experience as voiced by the mothers and aim to educate, inform and empower these women, as well as to involve them in decision-making as much as possible. To ensure professional competence and support at a high standard, ongoing skills development and advanced midwifery training should be of importance. Interventions aimed at improving interpersonal communication, connection and rapport between midwives and labouring women as well as allocation of enough midwives are also central to improve the quality of care. Hospital management needs to ensure the quality of the maternity care services, by providing a friendly, hygienic, private and safe environment for the mothers. Labour pain management is highly recommended. It is also recommended that further studies using qualitative interviews be conducted as women better articulate their feelings about pivotal life events such as childbirth, through opportunities to tell their story. In addition, a study using a follow-up questionnaire a few weeks after discharge may also prove to be valuable.

## Limitations

The study used a quantitative method, which limited the respondents’ opportunity to describe their experiences in detail. The study was conducted in two hospitals within the same district; therefore, this limits generalisation. Data were collected intensively within a short period of time; thus, information may have been obtained from subjects who may have been exposed to certain circumstances that existed in the research settings during that period, therefore influencing their experience of childbirth. The use of purposive sampling is a further limitation. Mothers responded to the questionnaire whilst still on hospital premises, and this might have possibly limited the disclosure of information and they might have given socially desirable responses to the researcher as they knew that she was a midwife. The missing data in some of returned questionnaires could have resulted in the limitation of important information as well as small amount of error in comparison with other countries as this study used a 5-point Likert scale.

## Conclusion

The experience of childbirth is described as multi-dimensional and subjective. Women have different views on what they consider to be a positive and satisfying birth experience, depending on both labour process and outcome experienced by them as individuals. Our study shows that both positive and negative experiences coexisted in all dimensions of the CEQ, with positive experiences being dominant in the majority of the respondents. Professional support dimension scored a high positive experience, whereas lack of participation, lack of privacy, poor sanitation and labour pains resulted in a negative experience. Thus, to gain a positive birth experience, the study suggests to ensure women’s own involvement, a safe, friendly environment and to be equipped with knowledge on the process of childbirth.

## References

[CIT0001] Adams, S.S., Eberhard-Gran, M. & Eskild, A., 2012, ‘Fear of childbirth and duration of labour: a study of 2206 women with intended vaginal delivery’, *BJOG: An International Journal of Obstetrics and Gynaecology* 119(10), 1238–1246. 22734617 10.1111/j.1471-0528.2012.03433.x

[CIT0002] Ahmar, E. & Tarraf, S., 2014, ‘Assessment of the socio-demographic factors associated with the satisfaction related to the childbirth experience’, *Open Journal of Obstetrics and Gynaecology* 4(10), 585–611. 10.4236/ojog.2014.410083

[CIT0003] Cook, K. & Loomis, C., 2012, ‘The impact of choice and control on women’s childbirth experiences’, *The Journal of Perinatal Education* 21(3), 158–168. 23730127 10.1891/1058-1243.21.3.158 PMC3392605

[CIT0004] Dencker, A., Taft, C., Bergqvist, L., Lilja, H. & Berg, M., 2010, ‘Childbirth experience questionnaire (CEQ): Development and evaluation of a multidimensional instrument’, *BMC Pregnancy and Childbirth* 10(1), 81–88. 10.1186/1471-2393-10-81 21143961 PMC3008689

[CIT0005] Downe, S., Finlayson, K., Oladapo, O., Bonet, M. & Gülmezoglu, A.M., 2018, ‘What matters to women during childbirth: A systematic qualitative review’, *PLoS One* 13(4), e0194906. 10.1371/journal.pone.0194906 29772012 PMC5957347

[CIT0006] Fair, C.D. & Morrison, T.E., 2012. ‘The relationship between prenatal control, expectations, experienced control, and birth satisfaction among primiparous women’, *Midwifery* 28(1), 39–44. 21458895 10.1016/j.midw.2010.10.013

[CIT0007] Fisher, J., Mello, M.C.D., Patel, V., Rahman, A., Tran, T., Holton, S. et al., 2012, ‘Prevalence and determinants of common perinatal mental disorders in women in low-and lower-middle-income countries: A systematic review’, *Bulletin of the World Health Organization* 90(2), 139–149. 10.2471/BLT.11.091850 PMC3302553 22423165

[CIT0008] Floyd, L., Coulter, N., Asamoah, S. & Agyare-Asante, R., 2014, ‘Women’s views and experience of their maternity care at a referral hospital in Ghana’, *African Journal of Midwifery and Women’s Health* 8(4), 168–175. 10.12968/ajmw.2014.8.4.168

[CIT0009] Hastings-Tolsma, M., Nolte, A.G. & Temane, A., 2018, ‘Birth stories from South Africa: Voices unheard’, *Women and Birth* 31(1), 42–50. 10.1016/j.wombi.2017.06.015 28711397

[CIT0010] Iravani, M., Zarean, E., Janghorbani, M. & Bahrami, M., 2015, ‘Women’s needs and expectations during normal labour and delivery’, *Journal of Education and Health Promotion* 4(1), 6. 10.4103/2277-9531.151885 25767817 PMC4355842

[CIT0011] Karlström, A., Nystedt, A. & Hildingsson, I., 2015, ‘The meaning of a very positive birth experience: Focus groups discussions with women’, *BMC Pregnancy and Childbirth* 15(1), 1–8. 10.1186/s12884-015-0683-0 26453022 PMC4600272

[CIT0012] Kordi, M., Bakhshi, M. & Tara, F., 2014, ‘The effect of continuous support during labour on labour progress in primigraviga women’, *The Iranian Journal of Obstetrics, Gynaecology and Infertility* 14, 7–14.

[CIT0013] Lozano, L.M., García-Cueto, E. & Muñiz, J., 2008, ‘Effect of the number of response categories on the reliability and validity of rating scales’, *Methodology: European Journal of Research Methods for the Behavioral and Social Sciences* 4(2), 73–79. 10.1027/1614-2241.4.2.73

[CIT0014] Lunda, P., Minnie, C.S. & Benadé, P., 2018, ‘Women’s experiences of continuous support during childbirth: A meta-synthesis’, *BMC Pregnancy and Childbirth* 18(1), 167. 10.1186/s12884-018-1755-8 29764406 PMC5952857

[CIT0015] Malatji, R. & Madiba, S., 2020, ‘Disrespect and abuse experienced by women during childbirth in Midwifery-Led Obstetric Units in Tshwane District, South Africa: A qualitative study’, *International Journal of Environmental Research and Public Health* 17(10), 3667. 10.3390/ijerph17103667 PMC7277802 32456063

[CIT0016] Maputle, M.S., 2018, ‘Support provided by midwives to women during labour in a public hospital, Limpopo Province, South Africa: A participant observation study’, *BMC Pregnancy and Childbirth* 18(1), 1–11. 10.1186/s12884-018-1860-8 29871607 PMC5989402

[CIT0017] Mensah, S., Mogale, R. & Richter, M., 2014, ‘Birthing experiences of Ghanaian women in 37th Military Hospital, Accra, Ghana’, *International Journal of Africa Nursing Sciences* 1(1), 29–34. 10.1016/j.ijans.2014.06.001

[CIT0018] Mohammad, K.I., Alafi, K.K., Mohammad, A.I., Gamble, J. & Creedy, D., 2014, ‘Jordanian women’s dissatisfaction with childbirth care’, *International Nursing Review* 61(2), 278–284. 10.1111/inr.12102 24762171

[CIT0019] Namujju, J., Muhindo, R., Mselle, L.T., Waiswa, P., Nankumbi, J. & Muwanguzi, P., 2018, ‘Childbirth experiences and their derived meaning: A qualitative study among postnatal mothers in Mbale regional referral hospital, Uganda’, *Reproductive Health* 15(1), 183. 10.1186/s12978-018-0628-y 30390685 PMC6215682

[CIT0020] Nilsson, L., Thorsell, T., Wahn, E.H. & Ekstrom, A., 2013, ‘Factors influencing positive birth experiences of first-time mothers’, *Nursing Research and Practice* 349124, 6 pages. 10.1155/2013/349124 PMC3793576 24175090

[CIT0021] Nystedt, A. & Hildingsson, I., 2014, ‘Diverse definitions of prolonged labour and its consequences with sometimes subsequent inappropriate treatment’, *BMC Pregnancy and Childbirth* 14(1), 233. 10.1186/1471-2393-14-233 25031035 PMC4105110

[CIT0022] Paudel, Y.R., Mehata, S., Paudel, D., Dariang, M., Aryal, K.K., Poudel, P. et al., 2015, ‘Women’s satisfaction of maternity care in Nepal and its correlation with intended future utilisation’, *International Journal of Reproductive Medicine* 2015, 783050. 10.1155/2015/783050 26640814 PMC4657080

[CIT0023] Renfrew, M.J., McFadden, A., Bastos, M.H., Campbell, J., Channon, A.A., Cheung, N.F. et al., 2014, ‘Midwifery and quality care: Findings from a new evidence-informed framework for maternal and newborn care’, *The Lancet* 384(9948), 1129–1145. 10.1016/S0140-6736(14)60789-3 24965816

[CIT0024] Sengane, M., 2013, ‘Mothers’ expectations of midwives’ care during labour in a public hospital in Gauteng’, *Curationis* 36(1), 1–9. 10.4102/curationis.v36i1.320 26697615

[CIT0025] Størksen, H.T., Garthus-Niegel, S., Vangen, S. & Eberhard-Gran, M., 2013, ‘The impact of previous birth experiences on maternal fear of childbirth’, *Acta Obstetricia et Gynecologica Scandinavica* 92(3), 318–324. 10.1111/aogs.12072 23278249

[CIT0026] Toohill, J., Fenwick, J., Gamble, J., Creedy, D.K., Buist, A. & Ryding, E.L., 2014, ‘Psycho-social predictors of childbirth fear in pregnant women: An Australian study’, *Open Journal of Obstetrics and Gynaecology* 4(9), 531–543. 10.4236/ojog.2014.49075

[CIT0027] Walker, K.F., Wilson, P., Bugg, G.J., Dencker, A. & Thornton, J.G., 2015, ‘Childbirth experience questionnaire: Validating its use in the United Kingdom’, *BMC Pregnancy and Childbirth* 15(1), 86. 10.1186/s12884-015-0513-4 25884191 PMC4396591

[CIT0028] Zhu, X., Wang, Y., Zhou, H., Qiu, L. & Pang, R., 2019, ‘Adaptation of the Childbirth Experience Questionnaire (CEQ) in China: A multisite cross-sectional study’, *PLoS One* 14(4), e0215373. 10.1371/journal.pone.0215373 31017927 PMC6481804

[CIT0029] Zitha, E. & Mokgatle, M.M., 2020, ‘Women’s views of and responses to maternity services rendered during labor and childbirth in maternity units in a semi-rural district in South Africa’, *International Journal of Environmental Research and Public Health* 17(14), 5035. 10.3390/ijerph17145035 PMC7400580 32668762

